# Supplementary material for: An Efficient Electroporation Protocol for the Genetic Modification of Mammalian Cells
Source: Front Bioeng Biotechnol. 2017 Jan 23;4:99. doi: 10.3389/fbioe.2016.00099 (PMC5253374; doi:10.3389/fbioe.2016.00099)
Supplement: Supplementary file 1 [file Data_Sheet_1.docx]

Supplementary Material

**An efficient electroporation protocol for the genetic modification of mammalian cells**

**Leonardo Chicaybam^1,6*^, Camila Barcelos^1*^, Barbara Peixoto^1^, Mayra Carneiro^1^, Cintia Gomez Limia^1^, Patrícia Redondo^2,3^, Carla Lira^4,5^, Flávio Paraguassú-Braga^4^, Zilton Vasconcelos^5^, Luciana Barros^1^, Martin Hernán Bonamino^1,6*^**

*** Correspondence:** Martin Hernan Bonamino: mbonamino@inca.gov.br

# Supplementary Figures and Tables

## Supplementary Figures





Figure S1: GFP expression mediated by SB transposon system in B16F10 cell line at d+3, d+7 and d+10 post nucleofection. Data are shown as mean ± SD from one experiment done in duplicate.





Figure S2: GFP expression mediated by SB transposon system in NIH3T3 cell line at d+3 and d+7 post nucleofection. Data are shown as mean ± SD from one experiment done in duplicate.





Figure S3: GFP expression mediated by SB transposon system in Ba/F3 cell line at d+3, d+7 and d+10 post nucleofection. Data are shown as mean ± SD from one experiment done in duplicate.





Figure S4: GFP expression mediated by SB transposon system in MDA231 cell line at d+3 and d+7 post nucleofection. Data are shown as mean ± SD from one experiment done in duplicate.





Figure S5: GFP expression mediated by SB transposon system in Jurkat cell line at d+3, d+7 and d+10 post nucleofection. Data are shown as mean ± SD from one experiment done in duplicate.





Figure S6: GFP expression mediated by SB transposon system in P815 cell line at d+3, d+7 and d+10 post nucleofection. Data are shown as mean ± SD from one experiment done in duplicate.





Figure S7: GFP expression mediated by SB transposon system in HeLa cell line at d+3, d+7 and d+10 post nucleofection. Data are shown as mean ± SD from one experiment done in duplicate.





Figure S8: GFP expression mediated by SB transposon system in 293T cell line at d+3 and d+7 post nucleofection. Data are shown as mean ± SD from one experiment done in duplicate.





Figure S9: GFP expression mediated by SB transposon system in Nalm-6 cell line at d+3, d+7 and d+10 post nucleofection. Data are shown as mean ± SD from one experiment done in duplicate.





Figure S10: GFP expression mediated by SB transposon system in HEL cell line at d+3, d+7 and d+10 post nucleofection. Data are shown as mean ± SD from one experiment done in duplicate.





Figure S11: GFP expression mediated by SB transposon system in MCF7 cell line at d+3 and d+7 post nucleofection. Data are shown as mean ± SD from one experiment done in duplicate.





Figure S12: GFP expression mediated by SB transposon system in K562 cell line at d+3, d+7 and d+10 post nucleofection. Data are shown as mean ± SD from one experiment done in duplicate.





Figure S13: GFP expression mediated by SB transposon system in A549 cell line at d+3 and d+7 post nucleofection. Data are shown as mean ± SD from one experiment done in duplicate.





Figure S14: GFP expression mediated by SB transposon system in MSC cells at d+3 and d+7 post nucleofection. Data are shown as mean ± SD from one experiment done in duplicate.





Figure S15: Long term stable expression of GFP from a SB based cassette conferring G418 resistance. NIH3T3 cells were electroporated with plasmid pT3-Neo EF1a-GFP and selected using G418 antibiotic (1 600ug/ml) for 10 days. After this period, G418 was withdrawn and GFP expression was accompanied for 5, 10 and 15 additional days. Data are shown as mean ± SD.


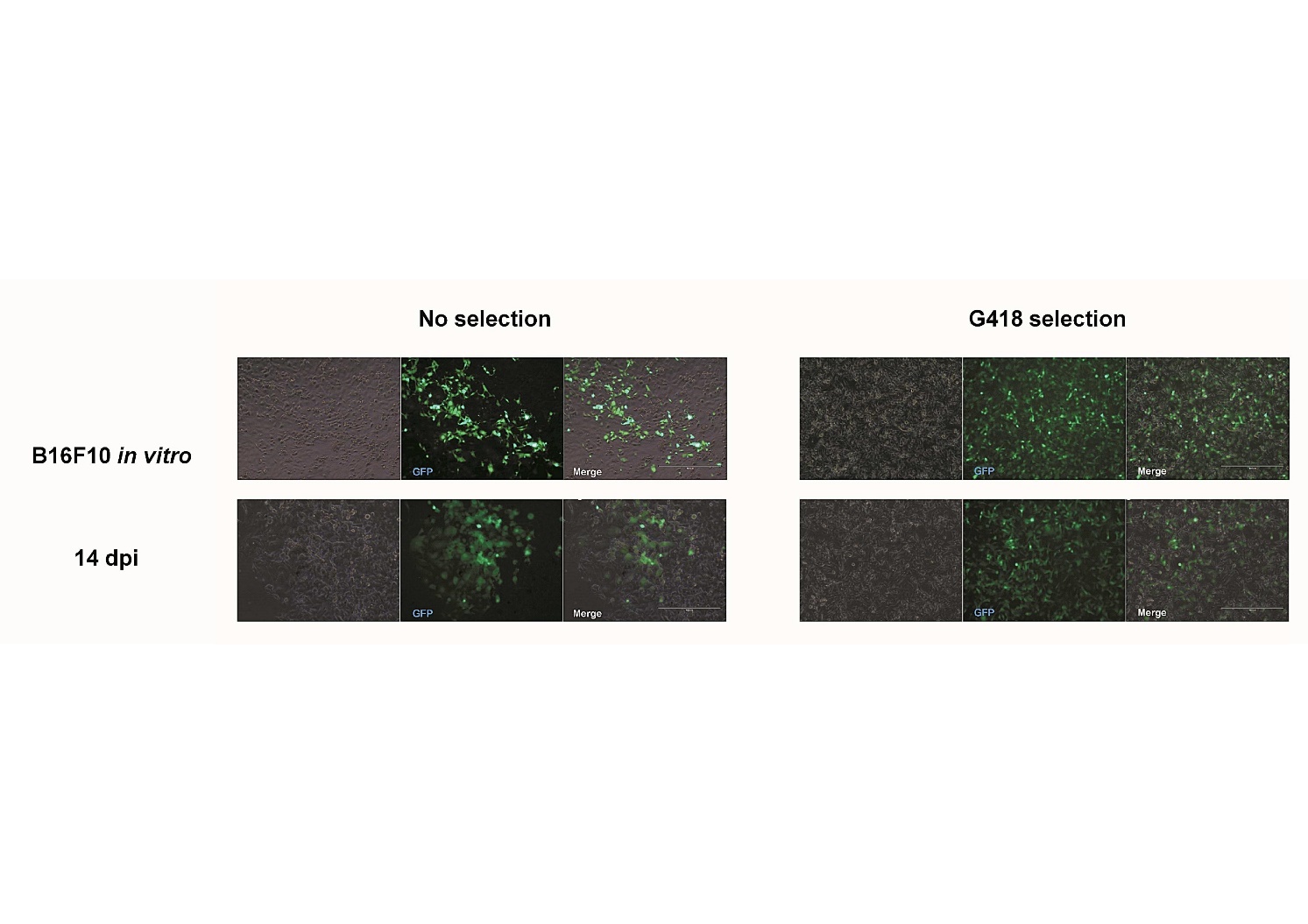


Figure S16: B16F10 cells can be enriched for GFP expression based on SB-Neo resistance cassette and retain high GFP expression after tumor formation in vivo. Fluorescent micrographs of B16F10 cells electroporated (buffer 1S, program P-020) with 10ug pT3-Neo-EF1a-GFP and kept in culture with or without selection with G418 (2000ug/mL). Cells were kept in vitro or recovered 14 days post injection (dpi) in C57Bl/6 mice. Bar = 400um


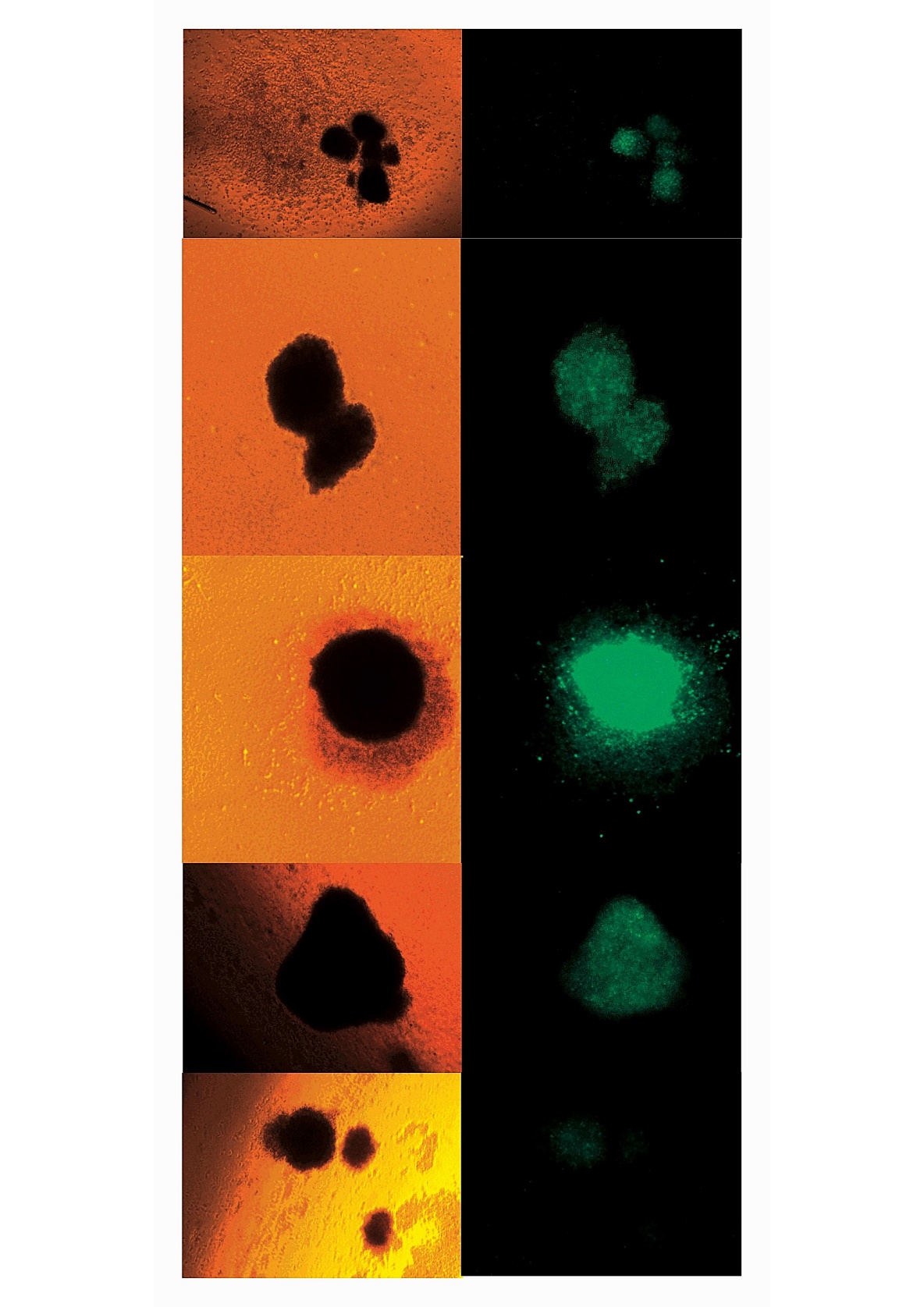


Figure S17: Representative images of CFU colonies generated from CD34+ cells electroporated with buffer 1SM and plasmids pT2-GFP and SB100x. Fluorescent micrographs were taken three weeks after induction of differentiation. All colonies shown represents BFU-E.


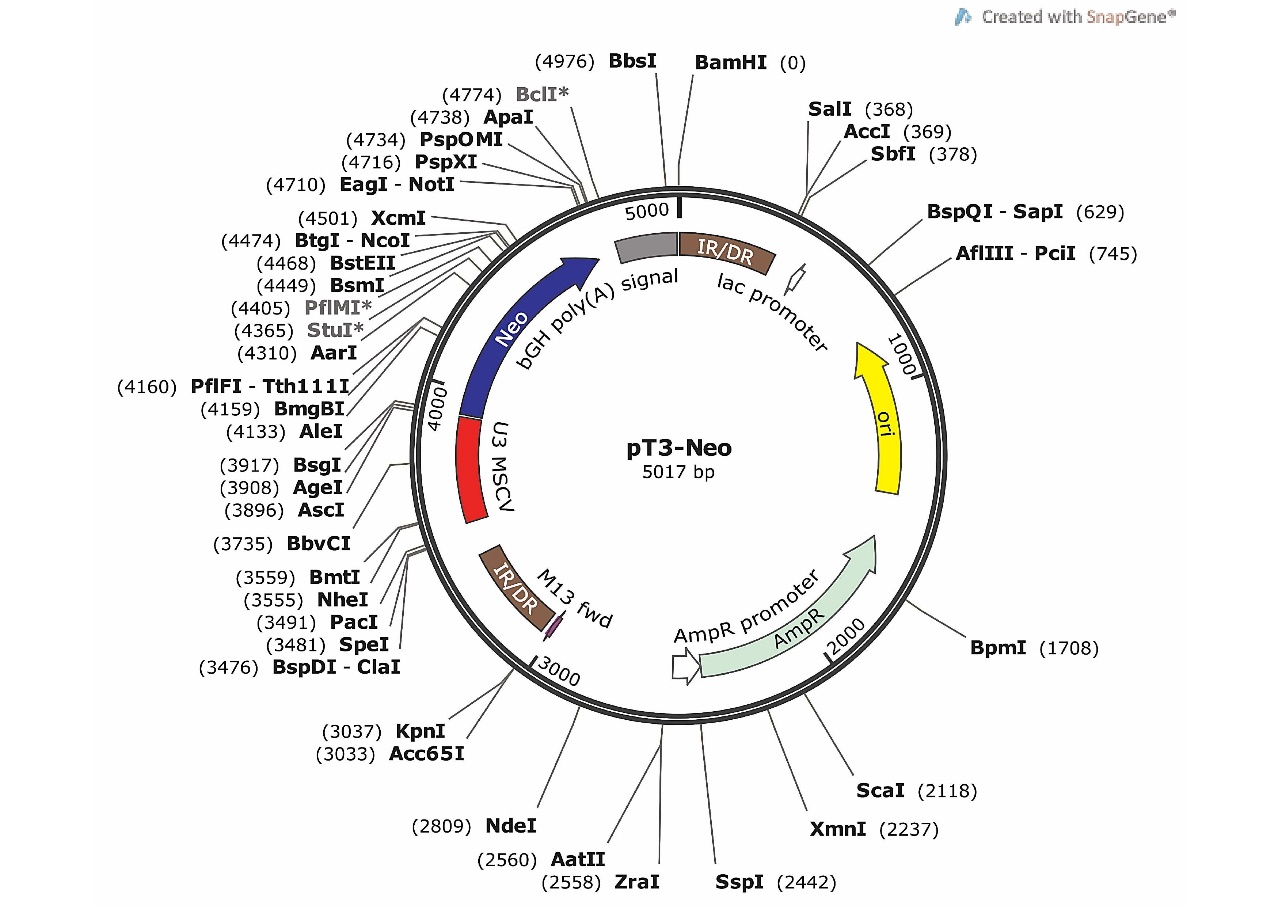


Figure S18: pT3-Neo plasmid map.


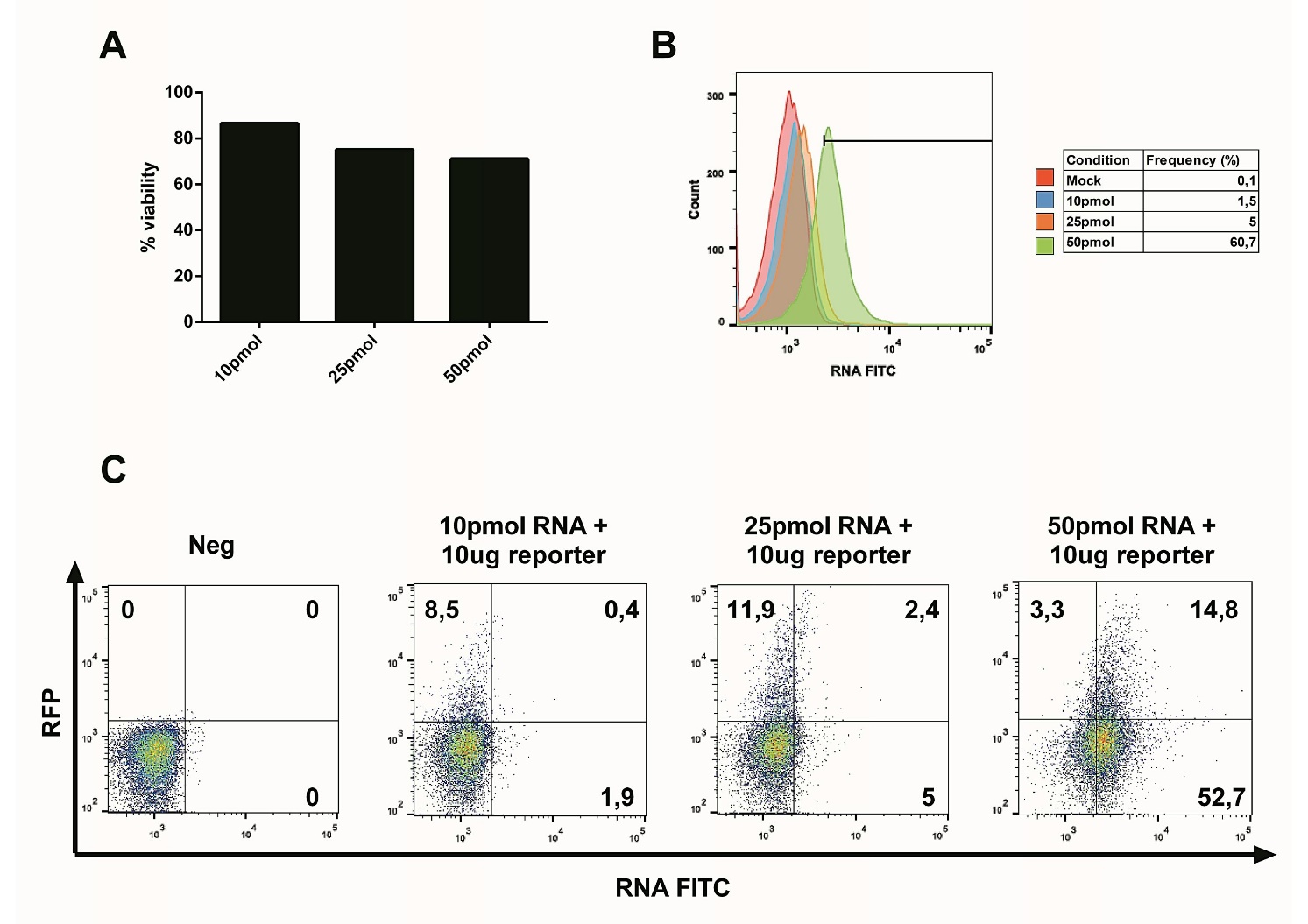


Figure S19: Co-electroporation of short RNA and plasmid in PBMCs. PBMCs were electroporated with varying amounts of a short RNA labeled with FITC (10pmol, 25pmol or 50pmol) in conjunction with 10ug of pRGS-CR using buffer 3P and program U-014. (A) After 24h, the cells were analyzed by flow cytometry and the viability (based in FSC/SSC morphology) was normalized against the mock electroporated control. (B) Histogram plot showing the expression of FITC-labeled RNA 24h after electroporation. Red = mock electroporated (negative control); Blue: 10pmol RNA; Orange: 25pmol RNA; Green: 50pmol RNA. (C) Dot plots showing the co-expression of RFP and RNA FITC 24h after electroporation. The numbers indicate the frequency in each gate. Data are derived from one experiment.

**1.2 Supplementary tables**

**Table S1: Culture conditions and origins of cell lines used in this study.**

| **Name** | **Culture conditions** | **Provided by** |
| --- | --- | --- |
| A549 | DMEM+10%SFB+1%L-Glu+1%Pen/Strep | Dr. Carlos Gil Ferreira, Brazilian National Cancer Institute, Brazil |
| 293T | DMEM+10%SFB+1%L-Glu+1%Pen/Strep | Dr. Elio Vanin, St. Jude Children's Research Hospital |
| B16/F10 | DMEM+10%SFB+1%L-Glu+1%Pen/Strep | Dr. João Viola, Brazilian National Cancer Institute, Brazil |
| HELA | DMEM+10%SFB+1%L-Glu+1%Pen/Strep | Dr. Marcelo Alex de Carvalho, Brazilian National Cancer Institute, Brazil |
| MCF7 | DMEM+10%SFB+1%L-Glu+1%Pen/Strep | Dr. Luize Lima, Brazilian National Cancer Institute, Brazil |
| MDA | DMEM+10%SFB+1%L-Glu+1%Pen/Strep | Dr. Maria Isabel Doria Rossi, Universidade Federal do Rio de Janeiro, Brazil |
| human MSCs | DMEM+10%SFB+1%L-Glu+1%Pen/Strep | Prepared from patients under informed consent |
| NIH3T3 | DMEM+10%SFB+1%L-Glu+1%Pen/Strep | Dr. João Viola, Brazilian National Cancer Institute, Brazil |
| Ba/F3 | RPMI+10%SFB+1%L-Glu+1% Pen/Strep+1% conditioned medium from XR63 (IL-3 source) | Dr. Andres Yunes, Centro Infantil Boldrini, Brazil |
| HEL | DMEM+10%SFB+1%L-Glu+1%Pen/Strep | Dr. Thomas Radimerski, Novartis Institutes for Biomedical Research, Basel, Switzerland |
| JURKAT | DMEM+10%SFB+1%L-Glu+1%Pen/Strep | Dr. João Viola, Brazilian National Cancer Institute, Brazil |
| K562 | DMEM+10%SFB+1%L-Glu+1%Pen/Strep | Dr. Thomas Radimerski, Novartis Institutes for Biomedical Research, Basel, Switzerland |
| NALM-6 | DMEM+10%SFB+1%L-Glu+1%Pen/Strep | Dr. Andrea Biondi, Fondazione M Tettamanti, Monza, Italy |
| P815 | DMEM+10%SFB+1%L-Glu+1%Pen/Strep | Dr. Adriana Bonomo, FIOCRUZ-RJ, Brazil |

Table S2: Buffers used in electroporation experiments.

| **Buffer** | **Composition** |
| --- | --- |
| 1M | 5mM KCl; 15mM MgCl2; 120mM Na2HPO4/NaH2PO4 pH7.2; 50mM Manitol |
|  |  |
| 1S | 5mM KCl; 15mM MgCl2; 120mM Na2HPO4/NaH2PO4 pH7.2; 50mM Sodium Succinate |
|  |  |
| 1SM | 5mM KCl; 15mM MgCl2; 120mM Na2HPO4/NaH2PO4 pH7.2; 25mM Sodium Succinate; 25mM Manitol |
|  |  |
| 2M | 5mM KCl, 15mM MgCl2; 15mM HEPES; 150mM Na2HPO4/NaH2PO4 pH7.2; 50mM Manitol |
|  |  |
| 2S | 5mM KCl, 15mM MgCl2; 15mM HEPES; 150mM Na2HPO4/NaH2PO4 pH7.2; 50mM Sodium Succinate |
|  |  |
| 3P | 5mM KCl, 15mM MgCl2; 90mM NaCl; 10mM Glucose; 0,4mM Ca(NO3)2; 40mM Na2HPO4/NaH2PO4 pH 7,2 |
|  |  |
| 3H | 5mM KCl, 15mM MgCl2; 90mM NaCl; 10mM Glucose; 0,4mM Ca(NO3)2; 20mM HEPES and 75mM Tris/HCl |
